# Using Intersectionality Theory as a Lens in a Perspectives on Aging Class

**DOI:** 10.1093/geroni/igab046.388

**Published:** 2021-12-17

**Authors:** Kelly Munly

**Affiliations:** Penn State Altoona, Altoona, Pennsylvania, United States

## Abstract

The presenter will discuss strategies for using intersectionality as a theoretical lens in her Perspectives on Aging class in order to support students to understand the relevance of aging studies—including health and social disparities in aging—for their contemporary lived lives, as well as for prior generations. With this relevance established, the class also examines the significance and justification for the development of policy, such as Social Security legislation, as well as the need for aging-related career areas. The presenter will discuss the application of key course resources, including research that looks at aging in historic contexts, as well as content highlighting the importance of Age Friendliness and the diversity of career areas to support Age Friendliness and more optimal aging experiences overall. Examining historic roots of aging-related experiences in social contexts creates an informative platform for understanding experiences of aging in society today.

